# Linear summation of outputs in a balanced network model of motor cortex

**DOI:** 10.3389/fncom.2015.00063

**Published:** 2015-06-05

**Authors:** Charles Capaday, Carl van Vreeswijk

**Affiliations:** ^1^Center for Neurophysics, Physiology and Pathology, Centre National de la Recherche Scientifique UMR 8119, Université Paris-DescartesParis, France; ^2^Department of Neurorehabilitation Engineering, University Medical Center Göttingen, Georg-August UniversityGermany

**Keywords:** motor cortex, balanced networks, linear summation, neural transfer functions, rate models, spiking models

## Abstract

Given the non-linearities of the neural circuitry's elements, we would expect cortical circuits to respond non-linearly when activated. Surprisingly, when two points in the motor cortex are activated simultaneously, the EMG responses are the linear sum of the responses evoked by each of the points activated separately. Additionally, the corticospinal transfer function is close to linear, implying that the synaptic interactions in motor cortex must be effectively linear. To account for this, here we develop a model of motor cortex composed of multiple interconnected points, each comprised of reciprocally connected excitatory and inhibitory neurons. We show how non-linearities in neuronal transfer functions are eschewed by strong synaptic interactions within each point. Consequently, the simultaneous activation of multiple points results in a linear summation of their respective outputs. We also consider the effects of reduction of inhibition at a cortical point when one or more surrounding points are active. The network response in this condition is linear over an approximately two- to three-fold decrease of inhibitory feedback strength. This result supports the idea that focal disinhibition allows linear coupling of motor cortical points to generate movement related muscle activation patterns; albeit with a limitation on gain control. The model also explains why neural activity does not spread as far out as the axonal connectivity allows, whilst also explaining why distant cortical points can be, nonetheless, functionally coupled by focal disinhibition. Finally, we discuss the advantages that linear interactions at the cortical level afford to motor command synthesis.

## Introduction

Microstimulation of a motor cortical point can evoke EMG output in several muscles. This can be quantified as a response vector. When two such points are simultaneously microstimulated, the evoked EMG vector is the linear sum of the EMG vectors evoked by microstimulation of each point on its own (Ethier et al., 2006). The result was unexpected and puzzling, given non-linearities in synaptic transmission and neural transfer functions. Additionally, the corticospinal transfer function—the relation between unit activity in the motor cortex (MCx) and motoneuron activation—is close to linear (Townsend et al., 2006; Capaday et al., 2013). This implies that the neural interactions within the motor MCx may be effectively linear. However, another possible explanation is that the distance between paired points studied by Ethier et al. (2006) was greater than the distance over which they interact. Several experimental observations make this suggestion unlikely. The distances between pairs of points studied by Ethier et al. (2006) ranged between 0.66 and 5.7 mm (mean = 2.65 mm, SD = 1.52 mm). The axon collaterals of motor cortical neurons extend up to 6–7 mm away from their soma and are studded with synaptic boutons all along their course (Capaday et al., 2009). Spiking at a motor cortical point ~0.4 mm in radius induces spiking in a surrounding area of ~1.5 mm in radius (Capaday et al., 2011). Thus, two points up to 3 mm apart, or perhaps more, share an overlapping territory. It follows that the distance between cortical points studied by Ethier et al. (2006) was on average within that over which they do interact. Taken together, these considerations suggest the more interesting possibility. The MCx circuitry may be wired to produce linear interactions between cortical points (Capaday et al., 2013).

**Figure 1:**
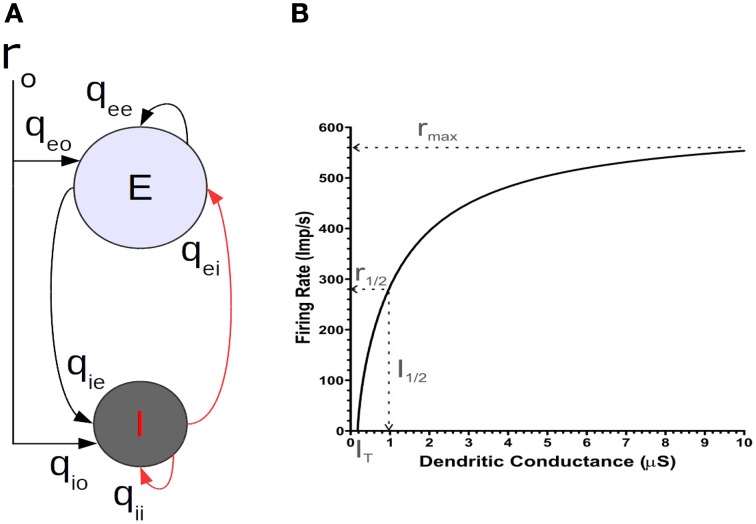
**(A)** The basic balanced neural network model of a single motor cortical point. **(B)** The f/I curve of a 2-C conductance based integrate and fire neuron model.

Balanced neural networks as originally proposed by van Vreeswijk and Sompolinsky (1996, 1998) involve a feedback dependent balance between synaptic excitation and inhibition such that, despite non-linear unit properties, the population output is a linear function of the input (Figure 1A). The population also responds to inputs with a time constant that is much shorter than that of the single units. In a balanced neural network the sum of the excitatory currents from external inputs, as well as from the activity of intrinsic circuit neurons, is balanced nearly exactly by the recurrent inhibitory currents. The basic idea of the balanced neural network is not unlike the principle used in operational amplifiers, where negative feedback of a portion of the output results in a device with linear input–output properties. The balanced network configuration also explains spike time variability. Spiking occurs at times when noise-like fluctuations generated by the dynamics of the circuitry exceed threshold. Spike timing is thus irregular and in fact asynchronous, even if the external input is temporally constant.

Here we present a model of MCx based on interconnected points that operate in a balanced state. The model explains how simultaneously active cortical points having non-linear unit properties can sum their outputs linearly and we discuss how this may simplify the synthesis of motor commands. We have chosen a level of mathematical description which avoids detailed, uncertain, or unavailable physiological measurements. The emphasis, nonetheless, is on keeping a close correspondence between the Mathematics and the Neurophysiology so as to obtain mechanistic explanations and testable physiological predictions. The model also allows us to understand the consequences of controlling the response gain of a cortical point by disinhibition and the mechanism that limits the spatial spread of activity. We also show that conduction and synaptic time delays do not destabilize the asynchronous state—i.e., time delays do not lead to synchronized activity. This extends the principles of balanced network operation.

## Materials and methods

There is considerable knowledge of the intrinsic, cable and repetitive firing properties of layer five neurons in the MCx (e.g., Crill and Schwindt, 1983; Stafstrom et al., 1984a,b). By contrast, little is known about the properties of neurons in other layers and in particular, those of inhibitory neurons. Similarly, the dynamics of synaptic transmission in the MCx *in vivo*, or *in vitro*, have not been investigated in sufficient detail. Here, we assume that synaptic transmission efficacy, that is the balance of synaptic facilitation and depression, is at steady-state over the time interval of movement duration (Abbott and Regehr, 2004). Taken together, the above considerations led us to use rate models as a simple description which allows us to understand the mechanisms of linear summation analytically. Rate models require fewer assumptions and many parameters, such as synaptic strengths, can be derived self-consistently (e.g., see Wilson, 1999). However, rate models cannot capture the fine structure of single unit spike timing and do not account for all types of spike synchronization. Therefore, we also simulated key aspects using networks of spiking model neurons.

In the models we will present, a cortical point consists of a population *e* of excitatory neurons and a population *i* of inhibitory neurons (Figure 1A). The two populations are reciprocally connected and each receives an external input representing a motor command. Additionally, each population is recurrently connected to itself. That is, the excitatory population is auto excited and the inhibitory one auto inhibited. Importantly, both neuron populations are driven by simultaneous excitatory and inhibitory currents, as has been shown in physiological experiments (e.g., Haider et al., 2006; Okun and Lampl, 2008). We will analyze the interactions of several such points that are fully interconnected by *e*-cell collaterals. As a first step, to uncover the basic mechanism by which the balanced network configuration leads to linearization of non-linear unit properties, we first consider in detail the operations of an isolated point.

### Basics of our rate model

We explain how temporal changes of firing rate depend on the input current. The firing rate *r_a_* of a given neuron population *a* (*a* = *e for the excitatory population, or i for the inhibitory population*) is given by

(1) $$\tau_{a}\frac{dr_{a}}{dt}\;=\;-r_{a}\;+\;f_{a}\left(I_{a}\right),$$

where τ_*a*_ is the time constant, *I_a_* is the total input current and *f_a_* (*I_a_*) represents the frequency/current (*f/I*) relation, or transfer function (e.g., see Shriki et al., 2003; Sompolinsky and White, 2004). We constrained the function *f_a_* (*I_a_*) of each population to that obtained *in vitro* from neurons of the cat motor cortex (Stafstrom et al., 1984b). This also constrains the range of synaptic connectivity strengths. Thus, the spike rate dynamics of a population of *n* recurrently connected neurons is reduced to a single system described by Equation (1) having the same effective synaptic strengths, and whose firing rate represents the population average. In our model, the activity of the excitatory neuron population represents the corticospinal output.

We considered the following two points to obtain the form of the *f_a_* (*I_a_*) relation. First, that the neurons constituting a given cortical point have an activation threshold and second, a non-linear transfer function. In this report, we use a transfer function motivated by our study of the neural mechanisms that allow modulation of a single neuron's firing rate gain by feedforward inputs (Capaday and van Vreeswijk, 2006). There we showed that, considering a two compartments (2-C) model, the firing rate gain can be modulated by a mixture of excitatory and inhibitory conductances in the dendritic compartment. Of equal importance, our analysis demonstrated that the transfer function of any spatially extended neuron model must be non-linear. To a first order approximation, the equation relating the synaptic input current at the soma *I_s_* and dendrite *I_d_* to the firing rate *r* of our 2-C model is given by

(2) $$r\;=\;\frac{1}{C_{m}\left(V_{T}\;-\;V_{r}\right)}\left[I_{S}\;+\;\frac{g_{c}}{g_{c}\;+\;g_{D}}I_{D}\right],$$

where *C_m_* is the membrane capacitance, *V_T_* the spiking threshold and *V_r_* the resting potential. Note how the dendritic synaptic current *I_D_* is attenuated by a factor *gc*/(*g_c_* + *g_D_*), where *g_c_* is the conductance coupling the dendritic compartment to the somatic compartment and *g_D_* is the dendritic conductance. Additionally, note that the firing rate given by Equation (2) is a decelerating function of the dendritic inputs (Figure 1B). We incorporate this effect in the *f_a_* (*I_a_*) relation of Equation (1) by a saturating transfer function, namely a first order Rushton–Naka function

(3) $$f_{a}\left(I_{a}\right)\;=\;{\left|\frac{\left(I_{a}\;-\;I_{T,\;a}\right)}{I_{1/\text{2},\;a}\;+\;\left(I_{a}-I_{T,\;a}\right)}r_{max,\;a}\right|}_{\text{+}.}$$

This function relates the firing rate *to the* input current, where *I*_1/2, *a*_ is the current required to reach the half-saturation rate *r_max, a_*/2 and *I_T, a_* is the current threshold. Note the half rectification symbol | |_+_, making *f_a_* (*I_a_*) a positive valued function. Following the result of Stafstrom et al. (1984b), *r_max, a_* was set to 250 Spk/s for both populations. From our analysis of the 2-C model, *I*_1/2_, *a*, the half-saturation current, was determined to be approximately 25 Spk/s. For the numerical simulations we present here, *I_T, a_* was set to zero because in the study by Ethier et al. (2006) all cortical points were stimulated at supra-threshold intensities. This choice is without loss of generality.

### Model of a single cortical point

Referring to Figure 1A, the net current *I_e_* driving the excitatory neurons is given by

(4) $$I_{e}\;=\;q_{eo}r_{o}\;+\;q_{ee}r_{e}-q_{ei}r_{i}.$$

While the net current driving the inhibitory population satisfies

(5) $$I_{i}\;=\;q_{io}r_{o}\;+\;q_{ie}r_{e}-q_{ii}r_{i}.$$

Here *q_ab_* is the charge injected in population *a* per spike in population *b*. The *q*-values thus represent the synaptic weights and have units of *charge/spike*. Consequently, the product *q_ab_r_a_* is a synaptic current (*charge/s*). Note that these synaptic weights represent the combined effects of all neurons in population *b* on a typical neuron in population *a*. Now, also take into account that only a few tens of presynaptic spikes can bring the postsynaptic neuron to threshold. Accordingly, because cortical neurons receive inputs from thousands of others, the magnitude of the synaptic currents *I_a_* (*a* = *e*, or i) will be large relative to the half-saturation current *I*_1/2, *a*_.

### Rate model of interacting cortical points

To understand quantitatively the interaction between motor cortical points we considered a fully interconnected three point model, with each point described by a balanced network (**Figure 5**). The connections between points are made by axon collaterals of the *e*-cell population at each point. The connections between the cortical points of the model correspond to the recurrent long range connections identified in the cat MCx (Capaday et al., 2009). Each point *k* receives an external input *r_o, k_* (*k* = 1, 2, or3). The output of the *e*-cell population at each point projects to the *e*-cell and *i*-cell populations of all other points. The inputs, *I_e, k_* and *I_i, k_*, to the excitatory and inhibitory cells, respectively, of point *k* are given by

(6) $$I_{a,\;k}\;={\text{ q}}_{\text{ao}}{\text{r}}_{\text{o},\text{ k}}\;+\;q_{ae}r_{e,\;k}-q_{ai}r_{i,\;k}\text{+}\sum_{l\neq k}w_{a,\;kl}r_{e,\;l}.$$

where *r_o, k_* is the rate of the external input to point *k, r_a, k_* is the rate of population *a* = *e*, or *i* at point *k*, and *w_a.kl_* is the strength of the intracortical connections from the *e*-cells of point *l* to the neurons in population *a* at point *k*. The first three terms on the right hand side of Equation (6) correspond to the within point currents of Equations (4) and (5). The terms under the summation symbol are the inputs from the other points. The synaptic strengths *w_a.kl_* decrease with the separation between points, in keeping with the monotonic decrease of the density of synaptic boutons projecting out of a motor cortical point (Capaday et al., 2009). By contrast, the local (i.e., within point) synaptic connection strengths (e.g., *q_ee_, q_ie_*, etc.) are identical for all points. Importantly, inhibition remains local, that is the *i*-cells do not project to other cortical points. This is also in keeping with a generally agreed upon organizational principle of cortical architecture (e.g., Bacci et al., 2005; Markram, 2010; Fino et al., 2013), notwithstanding that some inhibitory interneurons may have longer range projections (e.g., Tomioka et al., 2005). In addition, as previously suggested (Schneider et al., 2002) each point contains a population of what we term master inhibitory neurons (*MI*-neurons) whose function is to inhibit the local *i*-cells that feedback inhibit the local *e*-cells (**Figure 5**). These *MI*-neurons control, as we will show, the firing rate gain of a cortical point. The presence of specific *MI*-neurons, specialized to innervate other GABAergic interneurons, have been identified in the hippocampus (Freund and Antal, 1988; Acsády et al., 1996). Connections between various classes of inhibitory neurons is also a prominent feature in the neocortex (e.g., Fino et al., 2013). In particular, the recent finding that somatostatin expressing interneurons of the visual cortex do not inhibit each other, yet strongly inhibit all other inhibitory populations (Pfeffer et al., 2013), supports well the idea of *MI*-neurons. The addition of *MI*-neurons modifies the inhibitory feedback current onto the *e*-cell population by scaling the transfer function of the *i*-cell population according to the gain control mechanism described above. Thus, the maximal firing rate of the *i*-cell population *r_max, i_* in Equation (3) was simply scaled by a factor *G_i−cell_*, where 0 ≤ *G_i−cell_* ≤ 1.

### Spiking model of interacting points with delays

Rate models capture well the dynamics of neuronal populations, as long as the units within a population do not synchronize and the rate changes are not too rapid. For the single cortical point model rapid rate changes are not an issue because we are concerned with steady-state firing rates. However, our rate analysis cannot accurately determine whether the interaction between cortical points induces synchronous activity because of conduction delays between the points. Furthermore, as previously explained, rate models cannot capture the fine structure of single unit spike timing and do not account for all types of spike synchronization (e.g., see Roxin et al., 2005). To investigate these issues we did simulations of a network consisting of multiple points each of which contains populations of excitatory and inhibitory spiking neurons. The standard approach here would be to use networks of single compartment model neurons, either integrate and fire, or conductance based. But the *f/I* relationship of such cells is close to linear, particularly at higher rates, as already discussed. Thus, linearization by feedback via recurrent inhibitory connections is not meaningful in such networks. Consequently, we modeled the excitatory neurons as 2-C model neurons, consisting of a passive dendrite coupled to a soma having integrate and fire dynamics following Capaday and van Vreeswijk (2006). As shown there, these simple model neurons have strongly non-linear input–output curves when the input is a synaptic conductance change at the dendritic compartment, or at both the dendritic and somatic compartment. Recall that the firing rate of these 2-C model neurons is approximately given by Equation (2). Inhibitory interneurons tend to be electrotonically more compact. Accordingly we model the inhibitory cells as single compartment integrate and fire neurons.

Each cortical point consists of *N_e_* = 8000 excitatory and *N_i_* = 2000 inhibitory model neurons. All excitatory and inhibitory neurons in point *k* receive an external excitatory input proportional to *r_o.k_*, the firing rate of the external population that projects to the point. The neurons also receive recurrent inputs from, on average, 1000 excitatory and 1000 inhibitory neurons randomly chosen from the same point (van Vreeswijk and Sompolinsky, 1998). Additionally, the neurons receive inputs from excitatory cells in other points. The excitatory cells in point *k* receive inputs from, on average, *K_e_* (*k, l*) *e*-cells in point *l*, while for the inhibitory neurons that number is *K_i_* (*k, l*). This replicates the network connectivity of the rate model. To account for the finite conduction speed, these inputs arrive with a delay Δ*_kl_*. The distance between adjacent cortical points was 1 mm, consequently the conduction delay between points was set at 10 ms, as experimentally determined (Capaday et al., 2011).

The parameters of the 2-C excitatory neurons were based on electrophysiological measurements of cat layer five pyramidal cells *in vitro* (Stafstrom et al., 1984a,b). The somatic and dendritic membrane conductances were 0.06 and 0.12 μS, respectively. The coupling conductance between the two compartments was 0.06 μS. The somatic and dendritic membrane capacitances were 0.175 and 0.525 nF. The inhibitory neurons were modeled as single compartments having an input conductance of 0.1 μS and a membrane capacitance of 0.7 nF. For both model neurons the threshold was set at −60 mV, while the resting and reset potentials were set at −75 mV.

The synaptic inputs were modeled as instantaneous conductance changes. For the e-cells, 80% of inputs of all types were on the dendritic compartment. The reversal potentials of the excitatory and inhibitory synapses were set at 0 and −85 mV, respectively. A spike from the external inputs causes conductance changes of 10.66 and 1.18 nS in the *e*-cells and *i*-cells, respectively. Spikes from the *e*-cells in the network induce conductance changes of 5.33 and 1.18 nS, respectively, in the *e*-cell and *i*-cell population to which they are connected. The *i*-cells produce a conductance change per spike of 56.0 nS in the e-cells and 8.30 nS in the *i*-cells.

## Results

The results are presented as follows. In the first section we show that despite the non-linear *f/I* curves in the dynamic equations describing a single cortical point, the balanced network configuration produces nearly linear input–output curves. The reasons are explained analytically in the subsequent section. In the third section we demonstrate that the interactions between synaptically connected cortical points remain nearly linear for any combination of external or intracortical inputs, even when the strength of the local feedback inhibition is reduced. The response gain of a cortical point is also increased in the latter condition. Additionally, we show that by such an increase of its response gain, a cortical point which receives little or no effective input from a distant point can, nonetheless, be functionally coupled to it. An analytical treatment of the three point model explaining the mechanism of linear summation and its resilience to reduction of feedback inhibition is presented in the following section. Lastly, we demonstrate with the spiking model that conduction delays between cortical points does not lead to spike synchrony, or oscillations.

### Input–output characteristics of the single cortical point model

**Figure 2:**
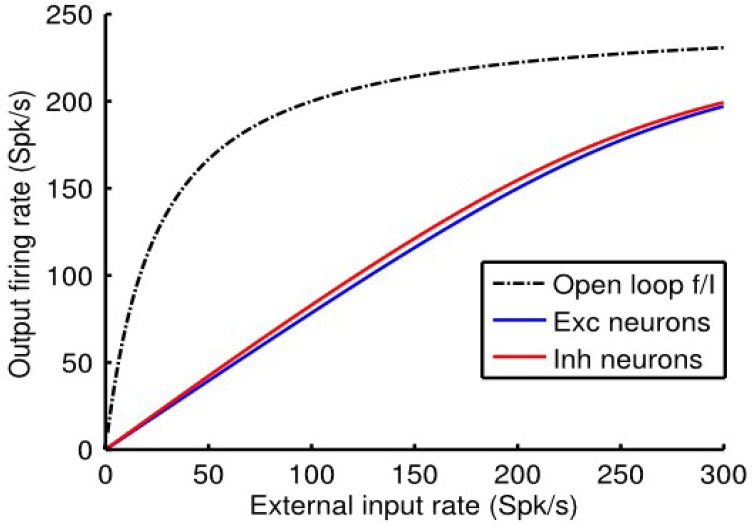
**Response of the single cortical point balanced network model to external input**. Note the near linear *f* (*I*) curves of the *e*-cell and *i*-cell populations. By contrast, the dashed black curve depicts the non-linear response of the output *e*-cell population in the open loop mode. Model parameter values were *q_ee_* = 0.67, *q_ei_* = −1.7, *q_ie_* = 1, and *q_ii_* = −2.

**Figure 3:**
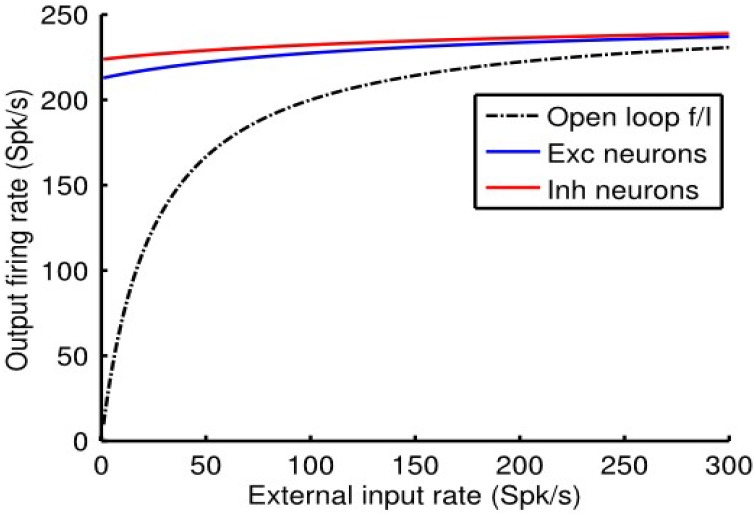
**Without feedback inhibition the cortical point is unstable, firing at near maximal rate for a minimal input**. Model parameter values as in Figure 2, except that *q_ei_* = 0 and *q_ii_* = 0.

We reiterate the fact that when the *e*-cell and *i*-cell populations are uncoupled (i.e., *q_ie_* = *q_ei_* = 0) and in the absence of any auto-feedback (i.e., *q_ee_* = *q_ii_* = 0) the open-loop *f_a_* (*I_a_*) curve of each population is non-linear (Figure 1B), following the Rushton–Naka transfer function Equation (3). Figure 2 shows how the steady-state output of the balanced network described by Equation (6) is related to the external input. The dashed curve is the open-loop *f_a_* (*I_a_*) curve of the *e*-cell, or *i*-cell, population shown for reference. The salient outcome resulting from the dynamic operation of the balanced network is that the *f_a_* (*I_a_*) curve of each population becomes markedly linear (Figure 2). The gain is essentially constant over the range of external inputs, but considerably lower at low rates (<50 Spk/s) compared to the open-loop gain. Setting all inhibitory connection strengths to zero (i.e., *q_ei_* = *q_ii_* = 0) results in a saturated near maximal output, even for a minimal input (Figure 3). For the *e*-cell population this is due to the strong recurrent excitatory feedback *q_ee_* unchecked by inhibition. For the *i*-cell population the effect is due to the resulting strong drive by the *e*-cell population and the absence of inhibitory auto-feedback. The model thus captures the instability characteristic of focal ictal activity produced by application of GABA_A_ receptor antagonists (e.g., Capaday et al., 2011). This observation and the fact that focal ictal activity can occur in an isolated cortical slab strongly support the idea that intracortical recurrent excitation must be strong and balanced by commensurate inhibition. The model is thus in the class of inhibition-stabilized networks (Vogels et al., 2005).

In summary, the dynamics of the balanced network linearized a non-linear system. The mechanisms underlying this transformation are dealt with in the following section.

### Analysis of the single cortical point model

Because the transfer functions of the excitatory and inhibitory populations are non-linear we would not, *a priori*, expect them to respond linearly with the external input. However, as we will show, the negative feedback between the populations and, importantly, the auto-inhibition of the inhibitory population produces linearization of the response. If the external population that drives the point fires with a constant rate *r_o_*, then after a transient, the rates of the excitatory and inhibitory populations reach a constant value. At this fixed point, the rates satisfy the following equations

(7) $$\begin{array}{c} r_{e}\;=\;{\left|\frac{I_{e}-I_{T,\;e}}{I_{1/\text{2},\;e}\;+\;I_{e}-I_{T,e}}r_{max,\;e}\right|}_{\text{+},}\\ r_{i}\;=\;{\left|\frac{I_{i}-I_{T,i}}{I_{1/\text{2},\;i}\;+\;I_{i}-I_{T,\;i\;}}r_{max,\;i}\right|}_{\text{+},} \end{array}$$

where the inputs, *I_e_* and *I_i_* are given by

(8) $$I_{e}\;=\;q_{eo}r_{o}\;+\;q_{ee}r_{e}-q_{ei}r_{i}\text{ and }I_{i}=q_{io}r_{o}\text{ + }q_{ie}r_{e}-q_{ii}r_{i}.$$

One can actually solve the set of Equation (7) analytically for *r_e_* and *r_i_*, but this involves finding the roots of a quartic equation whose form is not very revealing. Instead we will derive an approximate solution, which much more clearly explains how the linearization comes about. It also informs us on the conditions which the network parameters have to satisfy to get linearization of the response.

Under normal physiological conditions, the rates *r_e_* and *r_i_* should not be close to their maximum value for reasonable external input rates *r_o_*. Looking at Equation (7) we can deduce that this means that *I_e_* and *I_i_* should be small compared to *I*_1/2, *e*_ + *I_T, e_* and *I*_1/2_, *i* + *I_T, i_*, respectively. However, the synaptic strengths *q_ab_* are large. Consequently, the total external input *q_eo_r_0_*, the total recurrent excitation *q_ee_r_e_* and the total recurrent inhibition −*q_ei_r_i_* of the excitatory population will all be much larger in magnitude than *I*_1/2_, *_e_* + *I_T, e_*. The only way in which the net input *I_e_* can be sufficiently small is if the recurrent inhibition approximately cancels the feedforward input and recurrent excitation of the excitatory population. Thus, the rates should be such that

(9) $$q_{eo}r_{o}\;+\;q_{ee}r_{e}\approx q_{ei}r_{i}$$

Using the same argument for the inputs into the inhibitory population, we obtain a second equation that the rates should satisfy

(10) $$q_{io}r_{o}\;+\;q_{ie}r_{e}\approx q_{ii}r_{i}$$

So we have two requirements that have to be approximately satisfied. Combining the preceding two equations gives the approximate solutions for *r_e_* and *r_i_*, namely

(11) $$r_{e}\approx \frac{q_{eo}q_{ii}\text{​}-\text{​}q_{io}q_{ei}}{q_{ei}q_{ie}\text{​}-\text{​}q_{ee}q_{ii}}r_{o}\text{​}=\text{​}A_{e}r_{o},\text{ and }r_{i}\approx \frac{q_{eo}q_{ie}\text{​}-\text{​}q_{io}q_{ee}}{q_{ei}q_{ie}\text{​}-\text{​}q_{ee}q_{ii}}r_{o}\text{​}=\text{​}A_{i}r_{o}.$$

For a positive external input *r_o_* > 0, *r_e_* and *r_i_* should be positive. Thus, the weights *q_ab_* should be such that *A_e_* and *A_i_* are positive. The derivation makes the point that even though the transfer functions of the two populations are non-linear, the recurrent inputs make the response of the two populations approximately linear with the external input rate. In fact, in this approximation these rates do not depend on the properties of the open-loop transfer function at all, but only on the synaptic weights, *q_ab_*. This is because the large *q*-values force the network to an operating point where the total external input and excitatory recurrent feedback are roughly balanced by the recurrent inhibition in both the excitatory and inhibitory populations. In other words, at the operating point *I_e_* and *I_i_* will be dynamically zero. Additionally, Equations (11) imply that if we increase all *q_ab_*-values by the same factor (i.e., in proportion), the behavior of the differential Equations (1) does not change, but it can be shown that the linearization is improved.

We can use the fact that at the operating point of Equations (1) *I_e_* and *I_i_* are ≈ 0 to derive their linear approximation. To do this we calculate the Jacobian matrix *Df* of the system of Equations (1) evaluated at *I_e_* and *I_i_* ≈ 0, the result is

(12) $$Df\;=\;\left[\begin{array}{cc} \frac{r_{max,\;e}q_{ee}}{\tau_{e}I_{1/\text{2},\;e}} & \frac{-r_{max,\;e}q_{ei}}{\tau_{e}I_{1/\text{2},\;e}}\\ \frac{r_{max,\;i}q_{ie}}{\tau_{i}I_{1/\text{2},\;i}} & \frac{-r_{max,\;i}q_{ii}}{\tau_{i}I_{1/\text{2},\;i}} \end{array}\right].$$

Inspection of the Jacobian shows that the linear approximation has same synaptic weights as the non-linear system, except for a scaling factor γ_*a*_ = *r_max, a_*/τ*_a_I*_1/2_, *a* (*a* = *e*, or *i*). We can thus write the linear equivalent system as,

(13) $$\begin{array}{c} \tau_{e}\frac{dr_{e}}{dt}\;=\;-r_{e}\;+\;\gamma_{e}f\left(I_{e}\right),\\ \tau_{i}\frac{dr_{i}}{dt}\;=\;-r_{i}\;+\;\gamma_{i}f\left(I_{i}\right), \end{array}$$

where *f* (*I_e_*) and *f* (*I_i_*) are the linear functions given by Equations (8).

**Figure 4:**
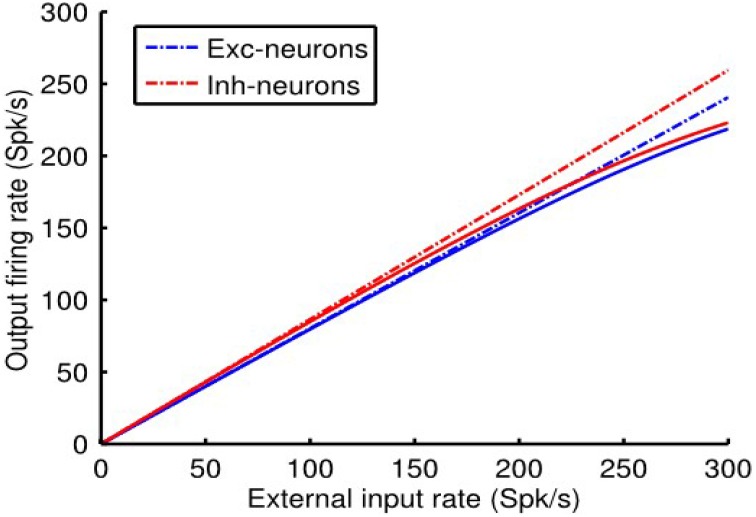
**Comparisons of linearization produced by the dynamics of the non-linear balanced network model vs. that of the linear system approximation**. Further details in the text. Model parameter values were *q_ee_* = 0.67, *q_ei_* = −1.7, *q_ie_* = 1, and *q_ii_* = −2.

Referring to Figure 4, it can be seen that the linear approximation closely follows the output of the non-linear balanced network model over the entire range of external inputs. As expected, however, there is a deviation at high firing rates because the linear approximation is not constrained by *r_max, a_*, whereas the non-linear system is. That is, in the linear approximation *r_max, a_* directly determines the scale factor γ_*a*_ = *r_max, a_/I*_1/2_, _*a*_. In the non-linear case, however, it is limiting because of the form of the Rushton–Naka function.

As a last point on the analysis of the single cortical point model we explain the conditions necessary to maintain stability and robustness. For the cortical point model to be stable, the eigenvalues λ_1_andλ_2_ of the Jacobian matrix must be negative. By considering the trace and determinant of the Jacobian matrix, it can be shown that stability requires *q_ie_q_ei_* > *q_ee_q_ii_* and that, γ*_i_q_ii_* > γ*_e_q_ee_*. In words, the product of the cross-feedback terms must be greater than the product of the auto-feedback terms and auto-inhibition should be sufficiently large compared to auto-excitation. This can be understood in neurophysiological terms as follows. Consider, for example, that *q_ee_* is very strong, this will drive the *e*-cells toward firing rate saturation. If *q_ie_* is weak, the *i*-cell population will not increase its firing rate sufficiently to counteract the high activity level of the *e*-cells, unless *q_ei_* is very strong. This explains why it is the product *q_ie_q_ei_* which is important. If instead, *q_ii_* is strong, then again the *e*-cell population will not receive sufficient inhibition unless either *q_ie_*, or *q_ei_* are strong. The two terms are once more linked in a multiplicative way. Note that this first requirement insures that the network activity is not oscillatory (i.e., the two populations firing in alternating bursts). The second stability requirement that γ*_i_q_ii_* >γ*_e_q_ee_* insures that the feedback inhibition of the *e*-cell population remains linear over the range of inputs. This prevents the activity level of either population from saturating.

Summarizing, the cortical point model responds nearly linearly to external inputs because the auto-inhibition of the inhibitory population results in a linear transfer function for this population and consequently linear feedback inhibition of the excitatory population.

### Input–output characteristics of coupled cortical points

The three point model afforded us the opportunity to determine the details of linear summation of simultaneously active cortical points and how this leads to linear summation of the evoked EMG vectors. It also allowed us to understand the consequences of controlling the response gain of a cortical point by disinhibition and the mechanism that limits spatial spread of activity. We begin with the details of linear summation.

Figure 6 shows the effects of activity at point-1 and point-2 on the output of point-3. Importantly, in this example point-3 is not driven by a direct external input, only by the activity of points with which it is connected. The firing rate response of point-3, to any combination of inputs from the other two points, lies on a nearly perfect plane. This demonstrates, as should be expected from the results of the previous section, that a given cortical point responds linearly to intracortical inputs (i.e., to activity from cortical points with which it is connected), in addition to external inputs. What may be less evident is that, because the long range connections strengths between points are weaker than the local within-point connections, a given cortical point operates on the strongly linear portion of its transfer function for intracortical inputs. Note in Figure 6 how the output of point-3 is limited to about 45 Spk/s for a maximal input from point-2 of 300 Spk/s. It also responds weakly, but also linearly, to inputs from the more distal point-1. Recalling that the points are fully interconnected, the response of point-3 also depends on its feedback onto points 1 and 2. To summarize, a given cortical point's gain for intracortical inputs is smaller than its gain for external inputs. This makes it operate on the strongly linear portion of the transfer function. In general, a linear system is defined as follows. If one obtains a response *r*_1_ to input *I*_1_ and a response *r*_2_ to input *I*_2_, then a linear system is strictly defined by the following relation

$$aI_{1}\;+\;bI_{2}\;=\;ar_{1}\;+\;br_{2}$$

for all pairs of inputs *I*_1_ and *I*_2_ and any combinations of the constants *a* and *b*. From the aforementioned it should be apparent that our three point model conforms to the definition of linearity.

The reader will recall that in both of the above examples the output is taken from a point which in physiological terms lies in the surround fringe of the activated point (i.e., points that are not directly activated). However, and importantly, it is the firing of all cortical points, whether directly activated or part of the indirectly activated fringe, that is transmitted down the corticospinal tract. Now, as stated in the introduction, since the corticospinal transfer function is essentially linear, it follows that the cortical firing rate vector $\vec{r}$ is related to the muscle activation vector $\vec{m}$ by the following simple model

(14) $$\vec{m}=W\vec{r}$$

where the matrix *W* contains the weighting coefficients. This model states that each cortical point has fixed connection weights to one or more motoneuron pools and that each motoneuron pool sums the inputs from one or more cortical points. The simple linear summation of muscle responses occurs precisely because the intracortical interactions are linear, this would not occur otherwise. In the following section we present the analysis that explains why the intracortical interactions are linear.

### Analysis of the coupled cortical points model

In the analysis of the effective linear response of the single point model we saw that, because the coupling strengths *q_ab_* are large, the network evolves to a state in which the recurrent inhibitory input almost perfectly cancels the external input and the recurrent excitation for both the excitatory and inhibitory populations. In the system of interacting cortical points the same principle applies. The system evolves to a state in which, at each cortical point *k*, the recurrent inhibition approximately cancels the external input, the recurrent excitation, as well as the inputs from other points. As in the single point model, this is true for the *e*-cell and *i*-cell populations.

Requiring that the recurrent inhibition balances the various excitatory inputs to the *e*-cells at point *k* yields, using Equation (6), that the inhibitory current approximately satisfies

(15) $$q_{ei}r_{i,\;k}=q_{eo}r_{o,\;k}\;+\;q_{ee}r_{e,\;k}\;+\;\sum_{l\neq k}w_{e,\;kl}r_{e,\;l},$$

while, from the balance of excitation and inhibition for the *i*-cells in population *k* we obtain approximately

(16) $$q_{ii}r_{i,\;k}=q_{io}r_{o,\;k}\;+\;q_{ie}r_{e,\;k}\;+\;\sum_{l\neq k}w_{i,\;kl}r_{e,\;l}.$$

Eliminating *r_i, k_* from these two equations we obtain

(17) $${\hat{q}}_{eo}r_{o,\;k}\;+\;{\hat{q}}_{ee}r_{e,\;k}\;+\;\sum_{l\neq k}{\hat{w}}_{e,\;kl}r_{e,\;l}\text{= 0},$$

with,

$${\hat{q}}_{eo}\text{​}=q_{eo}\text{​}-\;\frac{q_{ei}}{q_{ii}}q_{io},\;{\hat{q}}_{ee}\text{ ​}=\text{ ​}q_{ee}-\frac{q_{ei}}{q_{ii}}q_{ie},\text{ and }{\hat{w}}_{e,\;kl}\text{​ }=\text{ ​}w_{e,\;kl}\text{​}-\text{​}\frac{q_{ei}}{q_{ii}}w_{i,\;kl}.$$

This set of equations can be rewritten in vector notation as $\hat{q}$*_eo_*${\vec{r}}_{o}$ + *M*${\vec{r}}_{e}$ = $\vec{0}$, where ${\vec{r}}_{o}$ and ${\vec{r}}_{e}$ are vectors whose *k* − *th* elements are *r_o.k_* and *r_e, k_*, respectively. The effective connectivity matrix's elements are *M_kk_* = $\hat{q}$*_ee_* and *M_kl_* = $\hat{w}$*_e, kl_*, for *l* ≠ *k*. The rates of the excitatory populations are then simply given by ${\vec{r}}_{e}$ = −$\hat{q}$*_eo_M*^−1^${\vec{r}}_{o}$. Thus, effectively, the rate of the *e*-cell populations depend linearly on the external rates only. As a result, the response of the system to simultaneous activation of two or more points is just the sum of the responses to activation of each point individually.

### Local control of synaptic inhibition and i-cells

The more important insight gained from the coupled points model pertains to the consequences of local control of inhibitory synaptic strength and inhibitory feedback by the *MI*-neurons. When a cortical point is stimulated and a second cortical point disinhibited by focal iontophoretic release of a GABA_A_ antagonist, such as Bicuculline, the response is the same as if both points were stimulated together (Schneider et al., 2002; Ethier et al., 2006). Blocking of GABA_A_ receptors is equivalent to reducing *q_ei_* and *q_ii_* in the model. However, we realized that, as the linear approximation Equations (11) and (17) predict, changing the inhibitory synaptic strength parameters in proportion does not change the responsiveness of a cortical point to external or intrinsic inputs. Note that this is only approximately true for the non-linear system as can be appreciated from Figure 4. In any case, Equations (11) and (17) predict that, for example, to increase the responsiveness of a cortical point (i.e., its gain), *q_ei_* must be decreased more than *q_ii_*. The consequences of this are shown in Figure 7. In that example we demonstrate how local activity at point-1 affects activity at the more distal point-3 as the strength of the local inhibition *q_ei_* at point-3 is reduced. In this condition with the strength of local inhibition at point-3 set to a value of *q_ei_* = − 1.85, inputs from point-1 have a minimal effect on the output of point-3. As the strength of *q_ei_* is reduced, the firing rate gain of point-3 is increased. Note that because the inhibitory coefficient *q_ii_* was not changed, the *q_ei_/q_ii_* ratio decreased. (i.e., differentially controlled). The conclusion is that reduction of local inhibition allows for the functional coupling between points in a graded manner only if the *q_ei_/q_ii_* ratio is reduced. This has important implications for cortical Physiology in general and, in particular, for the mechanism by which iontophoretic release of GABA_A_ receptor antagonists allows cortical points to be coupled. These issues will be dealt with in the discussion.

**Figure 5:**
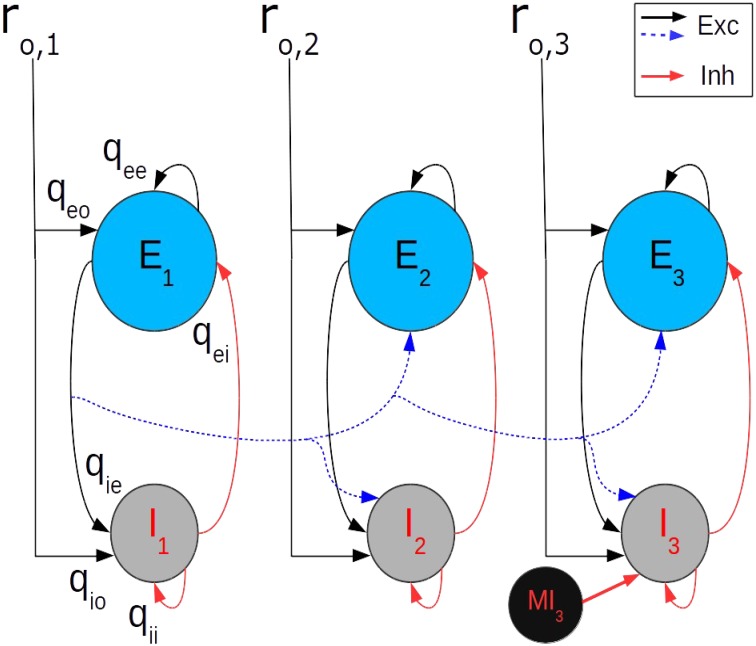
**Each point** ***k*** **of the three point model receives an external input r*****_o, k_***(***k*** **= 1, 2, 3)**. The output of the *e*-cell population (E) at each point projects to the *e*-cell and *i*-cell (I) populations of all other points, but the synaptic strengths decrease with separation. Importantly, inhibition remains local, i.e., the *i*-cells do not project to other cortical points. For clarity, only the projections of point-1 to point-2 and -3 are shown (blue dashed curves). The reader should keep in mind, however, that the three point model is fully recurrent. Likewise, the local (within point) synaptic connection strength labels (e.g., *q_ee_, q*_ie_, etc.) are shown only at point-1 and the MI-neuron only at point-3. The value of the within point synaptic connections strengths are identical for all points.

Reduction of inhibitory synaptic strength as described in the preceding section is non-specific, affecting both auto-inhibition of *i*-cells and feedback inhibition of *e*-cells and thus mimics GABA_A_ receptor blockade. To link these experimental observations to physiological function, we have suggested that the feedback inhibition of *e*-cells by *i*-cells may be controlled by *MI*-neurons (Schneider et al., 2002; Capaday, 2004; Capaday et al., 2013). The idea is that these interneurons are specialized at inhibiting the *i*-cell population thereby decreasing their response gain and consequently increasing the gain of the *e*-cells (Figure 5). In our model the *MI*-neuron population controls the firing rate gain *G_i−cell_* of the *i*-cell population to all inputs. This gain control mechanism is based on our 2-C neuron models as explained in the methods. In the example shown in Figure 8 we demonstrate how control of the local *i*-cell population by *MI*-neurons at point-2 modulates its response gain to inputs from point-1. The coupling strength between the two points was set at *q*_12_ = *q*_21_ = 0.2 and local inhibitory synaptic strengths, *q_ii_* and *q_ei_*, as in previous examples. Note that when *G_i−cell_* = 1 the *MI*-neurons exert no effect on the local *i*-cell population. As *G_i−cell_* is reduced, so too is the effectiveness of the inhibitory feedback on the *e*-cells and consequently the response gain increases. As can be seen in the figure, point-2 responds linearly to inputs from point-1, and with increasing gain as *G_i−cell_* is reduced. At a value nearing *G_i−cell_* ≈ 0.5, a deviation from linearity is apparent beyond *~*150 Spk/s. Thereafter, the response of point-2 follows an accelerating function, as in the example with *G_i−cell_* = 0.3. Thus, gain modulation by the *MI*-neurons maintains response linearity, but only over a limited range of 0.5 ≤ *G_i−cell_* ≤ 1. This effectively means no more than a gain doubling given the parameters of our model. The essential reason for this limitation is the maximum firing rate of the inhibitory population *r_max, i_*, limited to 300 Spk/s when *G_i−cell_* = 1.

**Figure 6:**
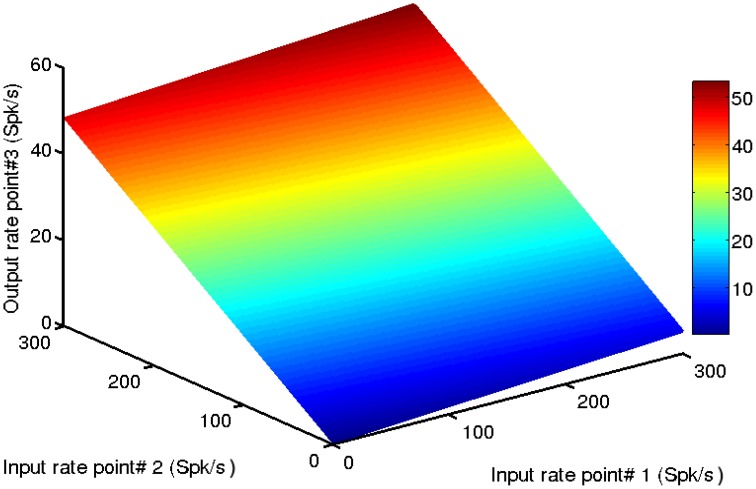
**A given cortical point responds linearly to inputs from surrounding points**. In this example point-3′s firing rate increases nearly linearly with inputs from point-1 and point-2. Note that inputs from point-1 have a minimal effect on the output of point-3 because the connections between these points are weak. Such a small effect may go unnoticed in an experiment. The connection strengths between the points were *w_e_*, _31_ = *w*_*i*, 31_ = 0.02 and *w*_*e*, 32_ = *w*_*i*, 32_ = 0.2. The color scale bar is in spikes per second (Spk/s).

**Figure 7:**
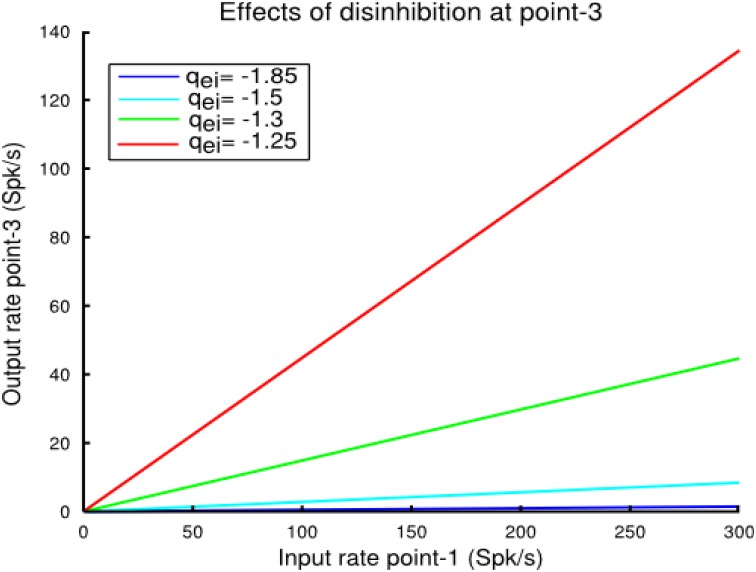
**In this example we show how local activity at cortical point-1 affects activity at a the more distal point-3 as inhibition strength at that point is reduced**. The coupling strength between the two points was set to *w*_*e*, 31_ = *w*_*i*, 31_ = 0.02 and *w*_*e*, 13_ = *w*_*i*, 13_ = 0.02. In this condition, with the strength of the local feedback inhibition set at a value *q_ei_* = −1.85, inputs from point-1 have a minimal effect on the output of point-3 (blue line). As *q_ei_* is reduced at point-3, the firing rate gain of this point is increased. Note, however, that the value of *q_ii_* was not changed. As explained in the text, functional coupling of two points by focal disinhibition only works when *q_ei_* and *q_ii_* are changed differentially, not in proportion.

**Figure 8:**
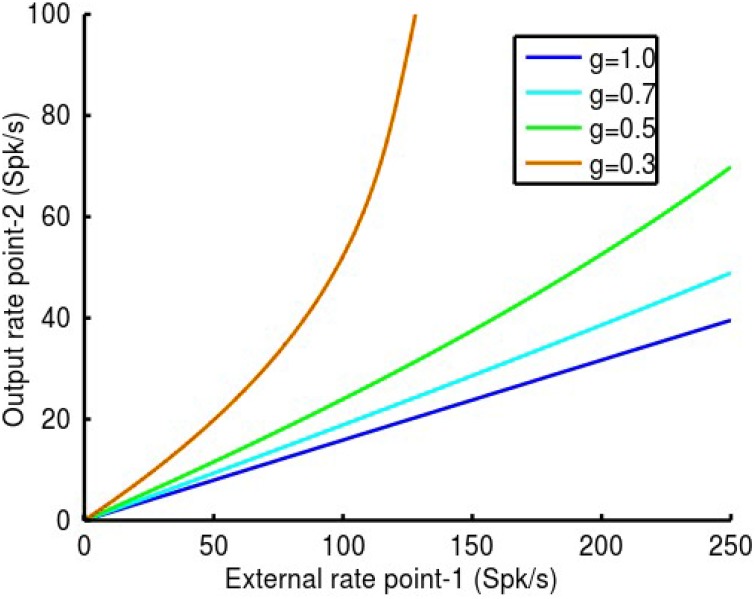
**In this example we show how** ***MI*****-neurons control of the local** ***i*****-cell population at point-2 modulates its response gain to inputs from point-1**. The coupling strength between the two points was set at *q*_12_ = *q*_21_ = 0.2 and local inhibitory synaptic strengths, *q_ii_* and *q_ei_*, as in previous examples. Note that when the *MI*-neurons exert no effect on the local *i*-cell s, *G_i-cell_* = 1. As *G_i-cell_* is reduced, so to is the effectiveness of the local inhibitory feedback and consequently the response gain increases. As can be seen in the figure, point-2 responds linearly to inputs from point-1, and with increasing gain as *G_i-cell_*is reduced. At a value nearing *G_i-cell_* ≈ 0.5, a deviation from linearity is apparent beyond ~150 Spk/s. Thereafter, the response of point-2 becomes an accelerating function, as in the example with *G_i-cell_* = 0.3. Thus, gain modulation by the *MI*-neurons maintains response linearity, but as expected, only over a limited range of 0.5 ≤ *G_i-cell_* ≤ 1. This effectively means not much more than a gain doubling.

We end this section by presenting the conditions necessary to maintain linearity when *MI*-neurons modulate the response gain of a cortical point. Considering Equation (7) in their linearized version, meaning that *I_a_* is much less than *I*_1/2, *a*_, we can write the steady-state firing rate of the *e*-cell population as

$$r_{e}\approx \frac{q_{eo}\left(q_{ii}\;+\;\alpha_{i}\right)-q_{io}q_{ei}}{q_{ei}q_{ie}\;-\;\left(q_{ee}-\alpha_{e}\right)\left(q_{ii}\;+\;\alpha_{i}\right)}r_{o},$$

where α_*e*_ = *I*_1/2, *e*_/*r_max, e_* and α_*i*_ = *I*_1/2, *i*_/*G_i−cell^r^max, i_*. One can appreciate that as *G_i−cell_* is reduced, α_*i*_ increases and consequently the gain term in Equation (18) increases. This linear approximation breaks down when the inhibitory rate *r_max, i_* approaches its maximum, as previously explained. The effect of the *MI*-neurons is to decrease the maximum firing rate of the *i*-cell population to *G_i−cell^r^max, i_*.

### Spiking model with conduction time delays

**Figure 9:**
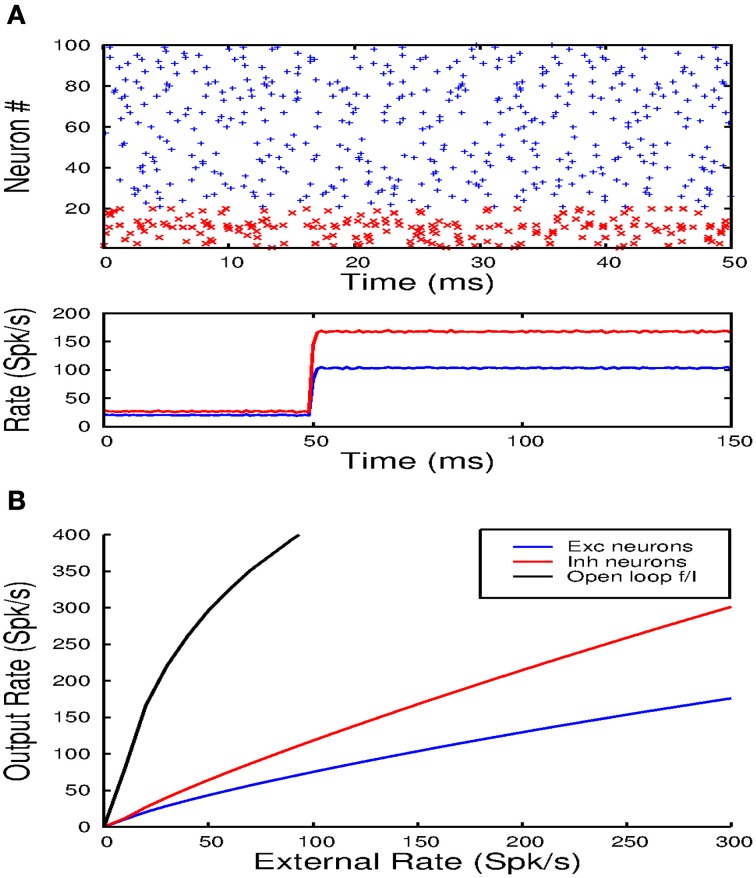
**(A)** The top panel shows a raster plot of the firing times of 80 excitatory and 20 inhibitory units over a time interval of 50 ms. Note that activity is irregular and asynchronous. The lower panel shows the averaged population response to a step input from which it can also be inferred that the population activity is not synchronized. **(B)** Shows how the balanced network configuration linearizes and reduces the gain of the population transfer function.

In these simulations of spiking networks, 80% of the synaptic inputs, of any kind, arrive on the dendritic compartment of the e-cells and 20% on the soma. Figure 9A shows the response of the network to a step input. The top panel shows a raster plot indicating the spike times of 80 excitatory neurons (blue) and 20 inhibitory cells (red) chosen at random. The lower panel shows the population averaged firing rate determined from the spike counts in 1 ms bins. The raster plot shows that the neurons fire irregularly and asynchronously. This is confirmed by the population averaged rate. After the onset of the step input, the average firing rates of the excitatory and inhibitory cells rapidly increase to a new steady-state value. When this value is reached, the number of spikes in each 1 ms time bin only varies by a small amount, indicating that activity is asynchronous. In Figure 9B the average firing rate in the steady-state is plotted against the strength of the step input for the excitatory (blue) and inhibitory (red) population. The open loop response for the excitatory cells (black) is also shown. Without local inhibitory feedback the excitatory rate first rises rapidly, after which the slope decreases progressively. With local inhibitory feedback the rates of both cell populations vary near linearly with the input rate. Due to the neuronal thresholds, the slope at the initial part of the curves is slightly higher. Nevertheless, as in the rate model, the feedback strongly linearizes the population output.

**Figure 10:**
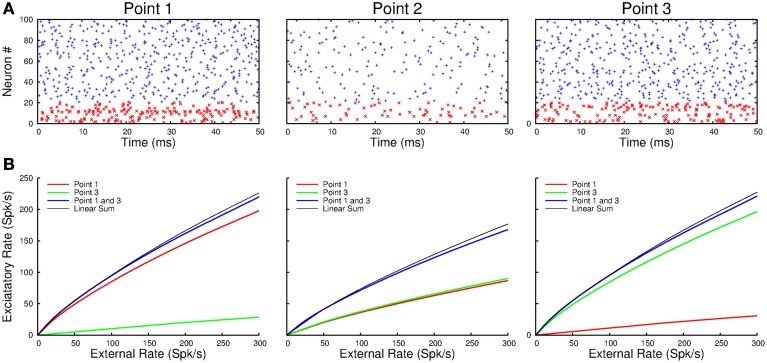
**(A)** The raster plots show the spiking activity at points-1 and -3 when they are simultaneously activated by external inputs. Note that point-2, which receives no direct input in this example, responds more weakly. **(B)** The population response at each point varies nearly linearly with the external input. Moreover, simultaneous activation of, in this example, point-1 and -3 results in responses at each of the points which is the sum of the responses to their separate activation.

Next we simulated a network of three interacting cortical points, with a distance of 1 mm and a conduction delay of 10 ms between adjacent points. Figure 10A shows a raster plot of the spiking activity of 80 excitatory and 20 inhibitory neurons in each of the points when point-1 and -3 are activated by step inputs. There is a strong response in points-1 and -3 and a weaker response in point-2. Nevertheless, despite the conduction delays, the activity is clearly asynchronous within and across points. Additionally, the neurons in the *e*-cell and *i*-cell populations fire irregularly. The three panels in Figure 10B show the firing rates of the e-cells in the three points as a function of the strength of the input when point-1 is activated (red), when point-3 is activated (green) and when point-1 and -3 are equally activated (blue). In all cases, the response of *e*-cell population at each of the points varies nearly linearly with the input strength. Moreover, at each of the points, the response when both point-1 and -3 are activated is very close to the sum of responses to activation of these points separately.

## Discussion

We have demonstrated a mechanism by which motor cortical points interact and respond to external command inputs linearly, despite non-linear neuron transfer functions. The balanced network configuration puts the motor cortical points in a linear negative feedback regime, as follows. At any given point, auto-inhibition of the *i*-cells effectively linearizes their transfer functions. Consequently, the corresponding *e*-cells receive linear negative feedback, which in turn linearizes their response. The resulting linear intracortical interactions yield linear summation of EMG output vectors, this would not occur otherwise. These conclusions were derived from analysis of a rate model. Additionally, numerical simulations of large populations of spiking neurons were done. These were connected according to the same balanced network principles. In this way, we demonstrated that conduction and synaptic time delays between cortical points does not lead to spike synchrony, or oscillations. We have also described how *MI*-neurons can control the response gain of a cortical point and thereby allow spatially separate points to be functionally coupled. However, the effectiveness of this mechanism appears limited to approximately a doubling of the gain, beyond which the response becomes non-linear. In summary, we have shown how the balanced network configuration may contribute to one important operational principle of MCx function, the linear summation of its outputs.

The results of a recent analysis of the behavior of excitatory and inhibitory neurons in various cortical areas, including the MCx, lend further support to our model (Dehghani et al., 2014). The units were separated into putative excitatory or inhibitory neurons based on their extracellular spike shape. The temporal activity of the two populations was shown to be strongly correlated in the way predicted by the balanced network configuration and this over multiple time scales ranging from a few milliseconds to tens of seconds.

### Novel features of the model its limitations and predictions

We introduced a network consisting of 2-C model neurons to capture an important physiological non-linearity, the relation between synaptic conductance and firing rate (Capaday and van Vreeswijk, 2006). A simple first order Rushton–Naka function describes well this relation derived from these 2-C models. The original formulation of the balanced network configuration by van Vreeswijk and Sompolinsky (1996) was based on binary units, contained no propagation or synaptic delays and the network is effectively a single cortical point. Despite the simplicity of binary units, the mathematical analysis of their interaction turned out to be involved (van Vreeswijk and Sompolinsky, 1998). Here we used a rate model which makes the mechanisms underlying linearization more apparent and the mathematics more approachable. For example, the conditions for stability of the network are more more easily derived and conform to intuition. These stability requirements make well-defined and testable experimental predictions that should further our knowledge of cortical circuitry. What's more, we have extended the balanced network principle to a network of interconnected cortical points whose interaction can be controlled by master inhibitory neurons. Lastly, the original balanced network formulation did not consider the effects of conduction and synaptic delays on network stability. Here we have shown that time delays do not destabilize the asynchronous state—i.e., time delays do not lead to synchronized activity. The reasons are that on the one hand, the delays to the *e*-cell and *i*-cell populations are equal and that on the other hand, the recurrent interactions within each point mitigate spike synchronization. This extends the principles of balanced network operation.

We had previously suggested that the muscle representations in different cortical points could be coupled together by selective excitation and release from inhibition, so as to produce movement related muscle activation patterns (Schneider et al., 2002). The results presented here show that the mechanism is plausible. Inhibition of the *i*-cells by the *MI*-neurons does increase the response of the *e*-cells allowing the disinhibited cortical point to be functionally coupled linearly with others. However, our analysis reveals that for the interaction to remain linear the gain increase must be modest, two to threefold in the present model. Whether in fact this mechanism is part of the process of motor command generation remains to be determined. A more refined model, that takes into account differential connectivity of *i*-cell population types, may accommodate a greater range of gain control while maintaining linearity. In any case, the range over which gain changes are possible need to be determined experimentally.

Our last point in this section is to explicitly state our model's most important prediction. The population response of any given motor cortical point will be the linear sum of its responses to separate inputs. For example, if a cortical point receives input from two different points, then the output of that point will be the linear sum of its response to each input on its own.

### Explanation of related experimental results

Focal iontophoresis of GABA_A_ receptor antagonists should reduce the auto inhibition of *i*-cells to the same extent as the feedback inhibition of the *e*-cells. Our model predicts that this should only have a minimal effect on the firing rate of the *e*-cell population. Consequently, coupling of cortical points by disinhibition should not occur. Yet the experimental evidence is clear, focal disinhibition of a cortical point allows it to be functionally coupled to the activity of a distant cortical point (Schneider et al., 2002). One possibility is that there is indeed a differential effect of GABA_A_ receptor antagonists on *q_ei_* and *q_ii_*, as we have modeled it here. Speculating, one may suggest that the binding affinities of receptor subtypes in the two populations may be different, or that the antagonists may have differential access to the receptors of each population. A second possibility arises from the stability requirement that γ*_i_q_ii_* > γ*_e_q_ee_*. At some concentration of GABA_A_ receptor antagonists, *q_ii_* will become smaller than *q_ee_*. The cortical point will then become unstable and discharge in synchronized periodic bursts. The consequences are different in each case. In the first case, the response at the disinhibited point will scale with the input as we have shown here. In the second case we have a triggered effect, the input triggers the instability and the point responds with a burst whose size is largely independent of the input strength. Only further experiments can unravel which of these mechanisms are involved. Nonetheless, the model predicts that during the phase of increased excitability when the neural activity is still asynchronous the disinhibited point will respond proportionately to inputs. By contrast, when the point is spontaneously bursting, the inputs will only reset the burst phase with relatively little effect on burst amplitude. Both possibilities can explain why the outputs of a microstimulated point and a second point made spontaneously active by focal disinhibition sum linearly (Ethier et al., 2006).

A third experimental observation explained by our model is why spike activity at one point does not spread over the full length of the extant connections (Capaday et al., 2011). Yet, upon disinhibition a more distal point can be recruited (Schneider et al., 2002). A given point has a small gain to distal inputs, consequently its response may be below threshold. When this point is disinhibited, its response gain increases sufficiently to respond to distal inputs with substantial spike activity leading to a motor output. It should be noted that this explanation is inconsistent with the long standing concept of surround inhibition, which would lead to sublinear summation.

### Functional advantage of linearity for motor command generation

What functional advantage does linear summation at the cortical level afford to the synthesis of motor commands? We propose that linear summation of the muscle synergies represented in different cortical points provides a simple prediction of the consequences of their simultaneous activation. This may simplify the generation of motor commands. Furthermore, assuming that the movement modules represented in the MCx are finite in number, interpolation is necessary to have a continuous space of possible movements. If the interactions within the MCx were non-linear, the interpolation process would be more challenging. We make no claim that the musculo-skeletal system is linear or controlled linearly, only that the motor cortical commands themselves are derived by linear combination. Results from a wide variety of approaches and experimental preparations show that a given class of movements, including locomotor movements and postural adjustments, are controlled by a small set of muscle synergies (reviewed by Bizzi and Cheung, 2013). Common to all the various theoretical formulations (e.g., D'Avella et al., 2006) is the linear combination of basic output modules. Neurophysiological evidence for the existence of neural circuits underlying these modules have been reported for pre-motoneuronal spinal networks and the MCx (Bizzi et al., 1991; Giszter et al., 1993; Schneider et al., 2001; Ethier et al., 2006; Graziano, 2006). What is more, the spinal modules and cortical points combine their respective outputs linearly (Mussa-Ivaldi et al., 1994; Ethier et al., 2006). More recently, it was suggested that the MCx selects and combines spinal modules temporally to produce the muscle activation patterns of reaching movements (Overduin et al., 2012).

### Epilogue

It may be suggested that linearization can be effected by two opposing non-linearities, one intracortical and one intraspinal. By considering established physiological and anatomical principles, we have provided evidence for an alternative explanation. Two consecutive linear stages account for the phenomenon.

### Conflict of interest statement

The authors declare that the research was conducted in the absence of any commercial or financial relationships that could be construed as a potential conflict of interest.
